# Novel molecular markers for the detection of methanogens and phylogenetic analyses of methanogenic communities

**DOI:** 10.3389/fmicb.2015.00694

**Published:** 2015-07-07

**Authors:** Lukasz Dziewit, Adam Pyzik, Krzysztof Romaniuk, Adam Sobczak, Pawel Szczesny, Leszek Lipinski, Dariusz Bartosik, Lukasz Drewniak

**Affiliations:** ^1^Department of Bacterial Genetics, Institute of Microbiology, Faculty of Biology, University of WarsawWarsaw, Poland; ^2^Laboratory of RNA Metabolism and Functional Genomics, Institute of Biochemistry and Biophysics, Polish Academy of SciencesWarsaw, Poland; ^3^Institute of Genetics and Biotechnology, Faculty of Biology, University of WarsawWarsaw, Poland; ^4^Department of Bioinformatics, Institute of Biochemistry and Biophysics, Polish Academy of SciencesWarsaw, Poland; ^5^Department of Systems Biology, Institute of Plant Experimental Biology and Biotechnology, Faculty of Biology, University of WarsawWarsaw, Poland; ^6^Laboratory of Environmental Pollution Analysis, Faculty of Biology, University of WarsawWarsaw, Poland

**Keywords:** methanogenesis, metagenomics, methanogenic consortia, *mcrB*, *mcrG*, *mtaB*, *mtbA*

## Abstract

Methanogenic *Archaea* produce approximately one billion tons of methane annually, but their biology remains largely unknown. This is partially due to the large phylogenetic and phenotypic diversity of this group of organisms, which inhabit various anoxic environments including peatlands, freshwater sediments, landfills, anaerobic digesters and the intestinal tracts of ruminants. Research is also hampered by the inability to cultivate methanogenic *Archaea*. Therefore, biodiversity studies have relied on the use of 16S rRNA and *mcrA* [encoding the α subunit of the methyl coenzyme M (methyl-CoM) reductase] genes as molecular markers for the detection and phylogenetic analysis of methanogens. Here, we describe four novel molecular markers that should prove useful in the detailed analysis of methanogenic consortia, with a special focus on methylotrophic methanogens. We have developed and validated sets of degenerate PCR primers for the amplification of genes encoding key enzymes involved in methanogenesis: *mcrB* and *mcrG* (encoding β and γ subunits of the methyl-CoM reductase, involved in the conversion of methyl-CoM to methane), *mtaB* (encoding methanol-5-hydroxybenzimidazolylcobamide Co-methyltransferase, catalyzing the conversion of methanol to methyl-CoM) and *mtbA* (encoding methylated [methylamine-specific corrinoid protein]:coenzyme M methyltransferase, involved in the conversion of mono-, di- and trimethylamine into methyl-CoM). The sensitivity of these primers was verified by high-throughput sequencing of PCR products amplified from DNA isolated from microorganisms present in anaerobic digesters. The selectivity of the markers was analyzed using phylogenetic methods. Our results indicate that the selected markers and the PCR primer sets can be used as specific tools for in-depth diversity analyses of methanogenic consortia.

## Introduction

**Figure 1:**
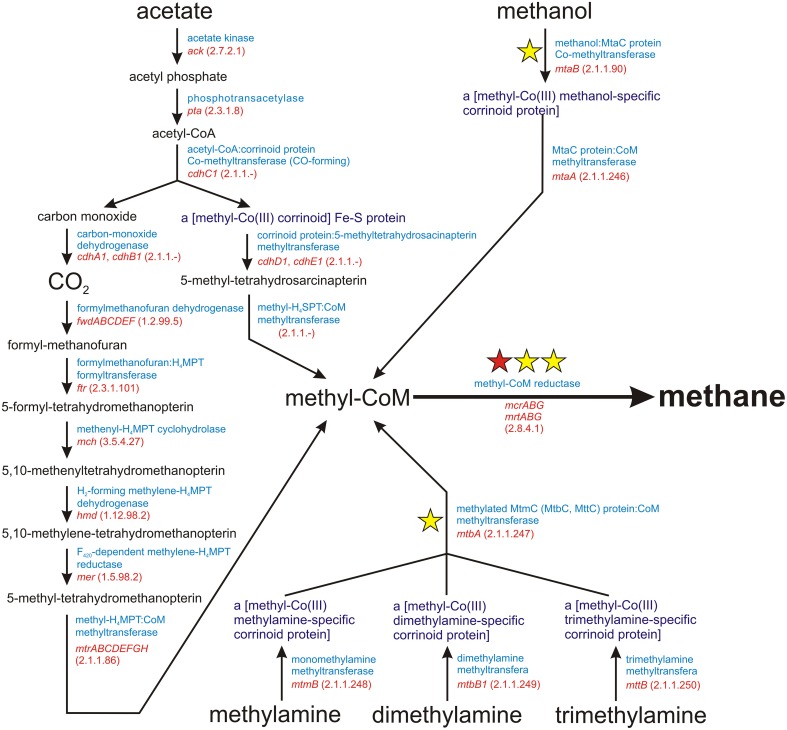
**Schematic diagram of the superpathway of methanogenesis**. E.C. numbers for particular enzymes are shown in parentheses. The red star indicates the *mcrA* gene encoding subunit α of a methyl-coenzyme M reductase I, which is commonly used as a molecular marker for the detection of methanogens. The yellow stars denote molecular markers developed in this study, for which sets of PCR primers were designed.

Methanogenesis is a metabolic process driven by obligate anaerobic *Archaea*. It is responsible for the production of over 90% of methane on Earth (Costa and Leigh, 2014). There are three main methanogenic pathways: (i) hydrogenotrophic methanogenesis using H_2_/CO_2_ for methane synthesis, (ii) acetoclastic methanogenesis, in which the methyl group from acetate is transferred to tetrahydrosarcinapterin and then to coenzyme M (CoM), and (iii) methylotrophic methanogenesis, using methyl groups from methanol and methylamines (mono-, di-, and trimethylamine) for the production of methyl-coenzyme M (Figure 1). The final step in all these pathways is common and involves the conversion of methyl-CoM into methane by methyl-coenzyme M reductase, an enzymatic complex that is present in all methanogens (Borrel et al., 2013) (Figure 1).

Methanogenesis is of great importance for biotechnology (e.g., fuel production) and environmental protection (methane emissions contribute to global warming) (Escamilla-Alvaradoa et al., 2012). Therefore, the process has been extensively studied (Gao and Gupta, 2007; Ferry, 2010; Yoon et al., 2013). Consequently, novel species representing particular groups of methanogens are regularly reported (e.g., Dridi et al., 2012; Garcia-Maldonado et al., 2015), and various tools for the genetic and bioinformatic analysis of methanogenic *Archaea* are being developed (e.g., Farkas et al., 2013; Zakrzewski et al., 2013).

Methanogenic *Archaea* form complex consortia which remain largely uncharacterized. Methanogens form close relationships with their syntrophic partners and require very specific environmental conditions for growth, so they have proven very difficult to cultivate in the laboratory (Sekiguchi, 2006; Sakai et al., 2009). Therefore, a number of culture-independent methods have been applied to examine methanogenic consortia: (i) community fingerprinting by denaturing gradient gel electrophoresis—DGGE (Watanabe et al., 2004), (ii) single strand conformation polymorphism—SSCP (Delbes et al., 2001), (iii) terminal restriction fragment length polymorphism—T-RFLP (Akuzawa et al., 2011), (iv) fluorescence *in situ* hybridization—FISH (Diaz et al., 2006), and (v) real-time quantitative PCR—qPCR (Sawayama et al., 2006). However, the most reliable approach for the characterization of methanogenic communities is high-throughput sequencing using either 454 pyrosequencing (e.g., Schlüter et al., 2008; Rademacher et al., 2012; Stolze et al., 2015) or Illumina sequencing technologies (e.g., Caporaso et al., 2011; Zhou et al., 2011; Kuroda et al., 2014; Li et al., 2014).

The most frequently used molecular marker for phylogenetic analyses in metagenomic studies, of methanogenic communities is the 16S rRNA gene. However, low specificity of the oligonucleotide primers employed means that they generate 16S rDNA amplicons for all *Archaea* (not only methanogens) whose DNA is present in the analyzed sample. In the search for a more specific molecular marker for methanogens, the gene encoding the α subunit of the methyl-CoM reductase (*mcrA*) was identified and primers were developed for its amplification (Springer et al., 1995; Lueders et al., 2001; Luton et al., 2002; Friedrich et al., 2005; Yu et al., 2005; Denman et al., 2007; Steinberg and Regan, 2009). Of these, primers designed by Luton et al. (2002), are probably the most extensively used in ecological studies, since they produce the lowest bias in amplifying *mcrA* gene fragments from a wide range of phylogenetically diverse methanogens (e.g., Juottonen et al., 2006).

Several studies have demonstrated that the phylogeny of methanogens based on 16S rDNA and *mcrA* markers is consistent, although greater richness is usually observed using the latter (Luton et al., 2002; Hallam et al., 2003; Bapteste et al., 2005; Nettmann et al., 2008; Borrel et al., 2013). Interestingly, Wilkins and coworkers showed that these two genes produce different taxonomic profiles for samples taken from anaerobic digesters, i.e., environments extremely rich in methanogens (Wilkins et al., 2015). Clearly, the characterization of methanogenic communities requires a systematic approach using reliable molecular markers.

In this study, we have developed a set of degenerate PCR primers for the amplification of genes encoding key enzymes involved in methanogenesis. Some of these represent an alternative to *mcrA* primers commonly used for metagenomic analyses of methanogens. These novel primers amplify fragments of other genes of the *mcr* cluster, i.e., *mcrB* and *mcrG* encoding subunits β and γ of methyl-CoM reductase, respectively. Moreover, we have identified appropriate molecular markers for methylotrophic methanogens, which are probably the least explored group of methanogenic *Archaea*. These primers amplify fragments of the genes *mtaB* (encoding methanol-5-hydroxybenzimidazolylcobamide Co-methyltransferase, which is responsible for the conversion of methanol to methyl-CoM) and *mtbA* (encoding methylated [methylamine-specific corrinoid protein]:coenzyme M methyltransferase involved in the conversion of methylated amines into methyl-CoM). The extended panel of molecular markers provided by these novel primer sets should permit a deeper insight into the complex phylogeny, biology, and evolution of methanogens.

## Materials and methods

### Standard genetic manipulations

PCR was performed in a Mastercycler (Eppendorf) using Taq DNA polymerase (Qiagen; with supplied buffer), dNTP mixture and appropriate primer pairs [Table 1 and additionally primer pairs S-D-Arch-0349-a-S-17/S-D-Arch-0786-a-A-20 for amplification of the variable region (V3V4) of the archaeal 16S rRNA gene (Klindworth et al., 2013), and MLf/MLr for *mcrA* gene amplification (Luton et al., 2002)]. PCR products of the methanogenesis-linked genes were purified by gel extraction, cloned using the pGEM®-T Easy Vector System (Promega) and transformed into *E. coli* TG1 (Stratagene) according to a standard procedure (Kushner, 1978). Standard methods were used for the isolation of recombinant plasmid DNA and for common DNA manipulation techniques (Sambrook and Russell, 2001).

**Table 1:** **Oligonucleotide primers (specific to *mcrB, mcrG, mtaB*, and *mtbA* genes) and PCR conditions**.

**Gene**	Name and sequence of the oligonucleotide primer^*^	**PCR product size**	PCR conditions^**^
*mcrB*	LMCRB: 5′- TWYCARGGHYTVAAYGC -3′ RMCRB: 5′- CCDCCDCCDCCRTARAT -3′	~392 bp	96°C–30 s; 56°C–30 s; 72°C–40 s 39 cycles
*mcrG*	LMCRG1: 5′-CAYCCDCCDYTNGADGARATGGA-3′ RMCRG1: 5′-TCRAACATYANWCCRTYYTCRTC-3′	~356 bp	96°C–30 s; 56°C–30 s; 72°C–35 s 39 cycles
*mtaB*	LMTAB: 5′- CARGCHAAYACYGCMATGTT -3′ RMTAB: 5′- CYTGDGGRTCYCKGTA -3′	~436 bp	96°C–30 s; 56°C–30 s; 72°C–40 s 39 cycles
*mtbA*	LMTBA: 5′- TTCTCCCTTGCMCAGCA -3′ RMTBA: 5′- ACWGGRTCVAGRTTWCC -3′	~413 bp	96°C–30 s; 55°C–30 s; 72°C–40 s 39 cycles

* *IUPAC code: A (adenine), C (cytosine), G (guanine), T (thymine), R (A or G), Y (C or T), W (A or T), K (G or T), M (A or C), D (A or G or T), H (A or C or T), V (A or C or G), N (A or C or G or T)*.

** *PCR conditions were specified for Taq DNA polymerase (Qiagen). The applicability of other (high fidelity) polymerases [i.e., Phusion High-Fidelity DNA Polymerase (Thermo Scientific) and KAPA polymerase (Kapa Biosystems)] was also confirmed*.

### Sample collection

Samples of microbial consortia involved in biogas production were collected from (i) the fermenter tank of an agricultural two-stage biogas plant anaerobic digester (AD) in Miedzyrzec Podlaski (Poland) and (ii) an effluent sludge tank from a one-stage wastewater treatment plant anaerobic digester (WD) at MPWIK Pulawy (Poland). In both cases, the samples were centrifuged (8000 g, 4°C, 15 min) and the pellets immediately stored in dry ice prior to DNA extraction.

### DNA extraction and purification

DNA was isolated from anaerobic digester samples using a modified bead beating protocol. 1 g of pellet material (containing solids and microorganisms) was resuspended in 2 ml of lysis buffer [100 mM Tris-HCl (pH 8.0), 100 mM sodium EDTA (pH 8.0), 100 mM sodium phosphate (pH 8.0), 1.5 M NaCl, 1% (w/v) CTAB] (Zhou et al., 1996). The cells were then disrupted by a 5-step bead beating protocol performed at 1800 rpm (4 × 15 s) and 3200 rpm (1 × 15 s) (MiniBeadBeater 8) using 0.8 g of zirconia/silica beads (ø 0.5 mm, BioSpec). After each round of bead beating the sample was centrifuged (8000 g, 5 min, 4°C), the supernatant retained, and the pellet resuspended in fresh lysis buffer. In addition, after the third round of bead beating, the samples were freeze/thawed five times. The supernatant from each round was extracted with phenol-chloroform-isoamyl alcohol [25:24:1 (vol)]. DNA was then precipitated with one volume of isopropanol, 0.1 volume of 3 M sodium acetate (pH 5.2), recovered by centrifugation at 13,000 g for 20 min, and the pellets washed twice with 70% (v/v) ethanol before resuspending in TE buffer.

The prepared DNA was purified to remove proteins, humic substances, and other impurities by cesium chloride density gradient centrifugation. The concentration and quality of the purified DNA were estimated using a NanoDrop 2000c spectrophotometer (NanoDrop Technologies) and by agarose gel electrophoresis. The applied method yielded highly pure DNA (A_260_/A_280_ = 1.8; A_260_/A_230_ = 1.9) suitable for metagenomic analysis.

### Library preparation and amplicon sequencing

PCR products were analyzed by electrophoresis on 2% agarose gels (1x TAE buffer) with ethidium bromide staining. The amplified DNA fragments from replicate PCRs were pooled and then purified using Agencourt AMPure XP beads (Beckman Coulter). Approximately 250 ng of each amplicon was used for library preparation with an Illumina TruSeq DNA Sample Preparation Kit according to the manufacturer's protocol, except that the final library amplification was omitted. The libraries were verified using a 2100 Bioanalyzer (Agilent) High-Sensitivity DNA Assay and KAPA Library Quantification Kit for the Illumina. Sequencing of amplicon DNA was performed using an Illumina MiSeq (MiSeq Reagent Kit v2, 500 cycles) with a read length of 250 bp.

### Designing oligonucleotide primers specific for *mcrB, mcrG, mtaB*, and *mtbA* genes

Data from the NCBI database were used to design degenerate primers to amplify *mcrB, mcrG, mtaB*, and *mtbA* gene fragments. A two-stage design strategy was employed. First, nucleotide sequences of genes annotated as *mcrB, mcrG, mtaB*, and *mtbA* were retrieved from the NCBI database. These sequences were then used as a query to recover additional gene sequences that were not annotated or were incorrectly annotated. Nucleotide sequences of particular genes were retrieved from genome sequences (completed and drafts) of methanogenic *Archaea* available on Jan 10th 2014. For each gene, multiple sequence alignments were prepared using ClustalW (Chenna et al., 2003) and MEGA6 (Tamura et al., 2013). Conserved regions within the obtained alignments were identified and used in the design of appropriate degenerate primers. Primer pairs with the lowest degree of degeneracy and producing amplicons not exceeding 500 bp were chosen. This size limit was imposed so that both 454 pyrosequencing and Illumina platforms could be used for amplicon sequencing.

*In silico* PCR with iPCRess (Slater and Birney, 2005) was done on dataset consisting of complete microbial genomes (5274 in total) obtained from NCBI database. We allowed for two mismatches per primer and required that both primers match and the product length is similar (±50 nucleotides) to expected length. The only exception was the set of *mcrG*-specific primers, that required allowance of 4 mismatches, due to bigger length of their sequences.

### Bioinformatic analysis of high-throughput amplicon sequencing data

For each selected protein family a reference set of sequences was assembled from the results of searches of the NCBI NR database with BLAST software (Altschul et al., 1997), using known archaeal members of each family as query sequences and an *E*-value of 0.001 as the threshold. These reference sets were not specifically curated to allow the presence of false positives such as proteins from *Bacteria* or *Eukarya*. We consider them false positives, as the process of methanogenesis is limited only to *Archaea*. A presence of the sequences more similar to bacterial homologs of marker proteins than to archaeal ones would indicate low specificity of the designed primers. We specifically screened for such a cases after phylogenetic placement of reads.

Paired-end reads were merged with FLASH (Magoc and Salzberg, 2011) and then mapped to reference sets using BLASTX, again with an *E*-value of 0.001 as the threshold. Translated sequences were extracted from the BLAST high scoring pairs (HSPs), and reads with no hits, containing stop codons (presumably generated by frameshifts) or sequences shorter than 30 amino acids were discarded. Therefore, for each primer pair, a reference set of known protein sequences was obtained, as well as a set of protein sequences derived from sequenced amplicons. The latter are referred as “inferred peptides” as they correspond to fragments of target proteins. The ratio of number of inferred peptides to number of all merged reads is the measure of primer sensitivity.

Sequences from the reference sets were aligned with MAFFT (Katoh and Standley, 2013) using default options. Based on these alignments, a maximum likelihood phylogenetic tree was constructed for each protein family using FastTree software (Price et al., 2009) with the Gamma20 model. Sequences inferred from reads were then merged with sequences from reference sets for each protein family and aligned with MAFFT as described above. The resulting alignment and the phylogenetic tree of reference sequences were used as the input to the Evolutionary Placement Algorithm, part of the RAxML package (Stamatakis, 2014). The reads were placed on the reference phylogenetic tree using the PROTGAMMAWAG substitution model. Placements were subsequently trimmed with guppy software (Matsen et al., 2010) using 0.01 as the minimal threshold mass from the leaf to the root. Results underwent guppy “fat” conversion to the PhyloXML file format (Han and Zmasek, 2009) and were then visualized using Archeopteryx software (Han and Zmasek, 2009). The visualization resulted in coloring branches that point to a node or a leaf to which reads were assigned in red. All other branches were colored in black.

Amplicons from 16S rDNA were processed differently. All sequence reads were processed via the NGS analysis pipeline of the SILVA rRNA gene database project (SILVAngs 1.2) (Quast et al., 2013). Using the SILVA Incremental Aligner [SINA SINA v1.2.10 for ARB SVN (revision 21008)] (Pruesse et al., 2012), each read was aligned against the SILVA SSU rRNA SEED and quality controlled (Quast et al., 2013). Reads shorter than 50 aligned nucleotides and reads with more than 2% ambiguities or 2% homopolymers, were excluded from further processing. In addition, putative contaminants and artifacts, and reads with low alignment quality (50 alignment identity, 40 alignment score reported by the SINA), were identified and excluded from downstream analysis.

The classification of each operational taxonomic unit (OTU) reference read was mapped onto all reads that were assigned to the respective OTU. This yielded quantitative information (number of individual reads per taxonomic path), within the limitations of PCR and sequencing technique biases, as well as multiple rRNA operons. Reads without any BLAST hits or those with weak BLAST hits, where the function “(% sequence identity + % alignment coverage)/2” did not exceed the value of 93, remained unclassified.

Raw sequences obtained in this study have been deposited in the SRA (NCBI) database with the accession number PRJNA284604.

## Results and discussion

### General diversity of *Archaea* in anaerobic digesters—16S rRNA and *mcrA* molecular marker analyses

**Figure 2:**
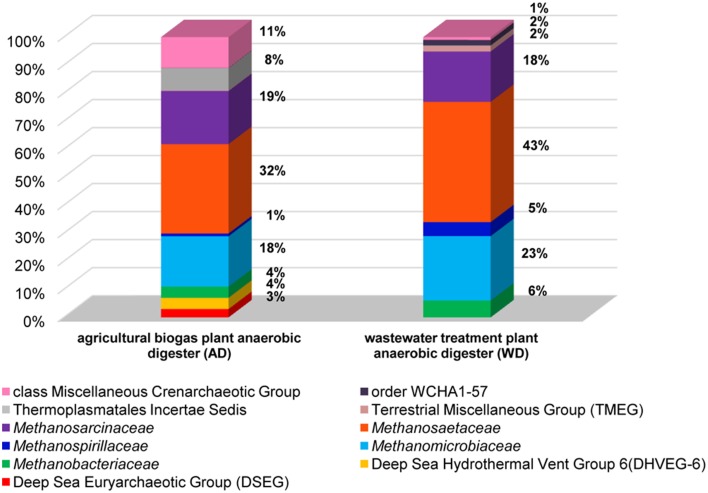
**Relative abundance of archaeal OTUs defined using the 16S rRNA gene hyper-variable region V3V4**. The bar chart shows the diversity of *Archaea* at the lowest reliable taxonomic level (where possible the default family is denoted in the key). AD, agricultural biogas plant anaerobic digester; WD, wastewater treatment plant anaerobic digester.

In the analyses performed in this study metagenomic DNA was extracted from two samples of microbial consortia involved in biogas production (and therefore reach in methanogens). For the description of the overall diversity of *Archaea* in the analyzed samples, 16S rDNA-specific primers were used (Klindworth et al., 2013). This analysis revealed that methanogens are dominant microorganisms in the studied anaerobic digesters (74% for AD and 95% for WD) and include representatives of four of the seven methanogenic orders (i.e., *Methanosarcinales, Methanomicrobiales, Methanobacteriales, Methanomassiliicoccales*). The most abundant methanogens in both digesters were *Methanosarcinales*, represented by the families *Methanosaetaceae* (~38%) and *Methanosarcinaceae* (~18%), followed by *Methanomicrobiaceae* (~20%) of the *Methanomicrobiales* order (Figure 2).

Abundant non-methanogenic *Archaea* such as Miscellaneous Crenarchaeotic Group (MCG) (11%) and *Halobacteria* (7%) represented by Deep Sea Euryarchaeotic Group (DSEG) and Deep Sea Hydrothermal Vent Gp 6 (DHVEG-6) were also detected in the AD sample (Figure 2). These groups are phylogenetically diverse and there is a little knowledge of their ecology and metabolism, however it seems that MCG archaeons are able to ferment wide variety of recalcitrant substrates (Kubo et al., 2012) and DSEG are positively correlated with putative ammonia-oxidizing *Thaumarchaeota* (Restrepo-Ortiz and Casamayor, 2013).

**Figure 3:**
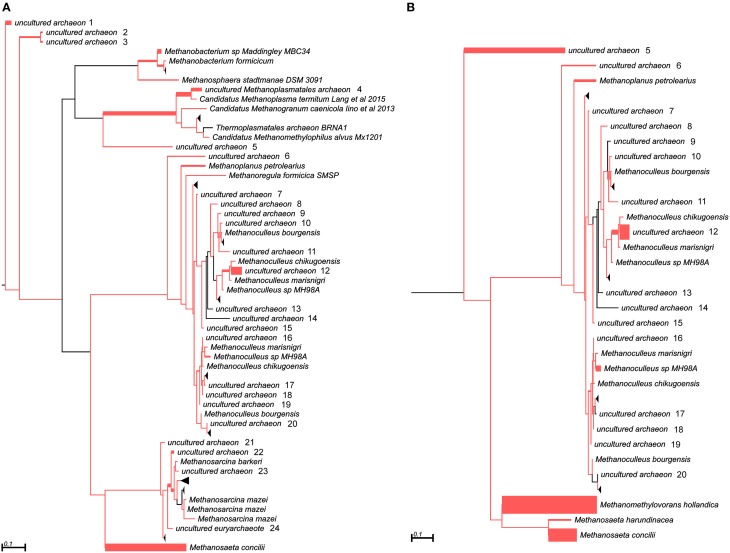
**Phylogenetic placement of *mcrA* amplicons from AD (A) and WD (B) samples**. The width of the red branches corresponds to the number of unique *mcrA* amplicon sequence reads in that particular branch (this can be either a leaf or node). The collapse of some branches (mapped to uncultured archaeons) to increase the clarity of the trees is indicated by a black triangle. Numbers next to the entries “uncultured archeon” indicate the same microorganism on both trees.

In addition to the 16S rRNA marker, the *mcrA* gene was used for taxonomic profiling of methanogenic communities in both digesters. The *mcrA* gene fragments amplified using primers MLf/MLr (Luton et al., 2002) were sequenced and analyzed. More than half of the sequences (57%) amplified from the AD sample were assigned to uncultured *Archaea*, belonging to the *Methanomassiliicoccales* (23%), *Methanomicrobiales* (13%), *Methanobacteriales* (11%) and *Methanosarcinales* (10%) orders (Figure 3), suggesting dominance of hydrogenotrophic methanogens over acetoclastic *Archaea*. The most abundant genera in AD were *Methanobacterium* sp. Maddingley MBC34 (11%) followed by *Methanosaeta concilli* (9%) and *Methanoculleus* spp. (4%) (Figure 3). Similarly in WD, the majority of the *mcrA* amplicons were classified as uncultured *Archaea* belonging to orders *Methanomicrobiales* (27%) and *Methanomassiliicoccales* (7%) (Figure 3), while at the genus level most of the methanogens were identified as *Methanometylovorans hollandica* (21%), *Methanosaeta concilli* (16%), *Methonoculleus* spp. (12%), or *Methanoplanus petrolearius* (3%) (Figure 3).

The results obtained for both marker genes (16S rRNA and *mcrA*) only partially overlapped, probably due to differences in primer affinities and variation in the gene copy numbers. This observation is in agreement with a previous report showing that these two marker genes generate different taxonomic profiles (Wilkins et al., 2015). Therefore, for a greater insight into the structure of methanogenic communities and to verify the obtained results, novel molecular markers specific for other methanogenesis-linked genes were developed.

### Development of *mcrB-, mcrG-, mtaB*-, and *mtbA*-specific primers

For the design of degenerate primers specific for the *mcrB, mcrG, mtaB*, and *mtbA* genes, sequences were retrieved from the NCBI database [36 nucleotide sequences for *mcrB* (Figure S1), 61 for *mcrG* (Figure S2), 26 for *mtaB* (Figure S3) and 13 for *mtbA* (Figure S4)]. The *mcrG* gene turned out to be highly variable, which hampered primer design. Therefore, phylogenetic analysis was performed to distinguish conserved clusters among the analyzed *mcrG* genes. Two groups of *mcrG* sequences were distinguished: (i) MCR_G1 (grouping 35 *mcrG* genes of *Methanobacterium* spp., *Methanobrevibacter* spp., *Methanocaldococcus* spp., *Methanococcus* spp., *Methanothermobacter* spp., *Methanothermococcus* spp., *Methanothermus* spp., *Methanotorris* spp., *Methanosphaera* spp.), and (ii) MCR_G2 (grouping 26 *mcrG* genes of *Methanocella* spp., *Methanococcoides* spp., *Methanocorpusculum* spp., *Methanoculleus* spp., *Methanohalobium* spp., *Methanohalophilus* spp., *Methanolobus* spp., *Methanoplanus* spp., *Methanopyrus* spp., *Methanoregula* spp., *Methanosalsum* spp., *Methanosarcina* spp., *Methanospirillum* spp., *Methanosphaerula* spp.) (Figure S5). The nucleotide sequences of *mcrG* genes from particular groups were then used to design specific primer pairs.

For the subsequent functional analyses, 28 primers were selected for synthesis, including 6 for *mcrB*, 9 for *mcrG* and *mtaB*, and 4 for *mtbA*. The initial PCRs were performed with all primer pairs and DNA samples from the AD and WD fermenters as templates. The primer pairs giving the strongest amplification products of the expected size were selected for further analysis. The PCR products were cloned in vector pGEM-T Easy and then inserts of five random clones from each experimental set were sequenced using the sequencing primer M13 Reverse. The BLAST analysis of the resulting sequences revealed the specificity of each primer pair. At this stage, all primers designed for amplification of the *mcrG* genes of MCR_G2 group methanogens were rejected due to low specificity. Based on those analyses and taking into account the amplification yield, four primer pairs were selected and the optimal PCR conditions were determined (Table 1). Primer pairs specificity was also initially confirmed by *in silico* PCR analysis using 5274 complete microbial genomes (Table S1).

Since the panel of primers developed in this study was designed to be used in the high-throughput amplicon sequencing analysis of methanogenic communities, their selectivity was tested in the high-throughput sequencing experiments.

### Analysis of the selectivity of the *mcrB*- and *mcrG*-specific primers

DNA fragments were amplified using the developed primer pairs with template DNAs isolated from the anaerobic reactors AD and WD. The raw sequence data obtained from Illumina sequencing were processed and analyzed (Table 2).

**Table 2:** **Summary of bioinformatic analysis of sequenced *mcrA, mcrB, mcrG, mtaB*, and *mtbA* amplicons**.

Sample^*^	**Number of paired reads**	**Number of merged reads**	Number of inferred peptides^**^	**Primer sensitivity (% of correct product)**
mcrA_AD	17,365	12,816	11,931	93
mcrA_WD	9277	4318	2572	59
mcrB_AD	32,094	23,188	21.939	94
mcrB_WD	50,485	40,242	25,035	57
mcrG_AD	42,185	29,330	21,988	74
mcrG_WD	34,945	28,660	18,272	63
mtaB_AD	26,500	20,753	19,163	92
mtaB_WD	36,148	15,293	13,231	86
mtbA_AD	33,770	22,852	10,027	43
mtbA_WD	31,601	19,150	10,961	57

* *AD, agricultural biogas plant anaerobic digester; WD, wastewater treatment plant anaerobic digester*.

** *Inferred peptides number denote how many peptides that are sufficiently long and similar to a target protein can be extracted from the reads. Percent of correct product is the ratio between number of peptides and number of reads*.

**Figure 4:**
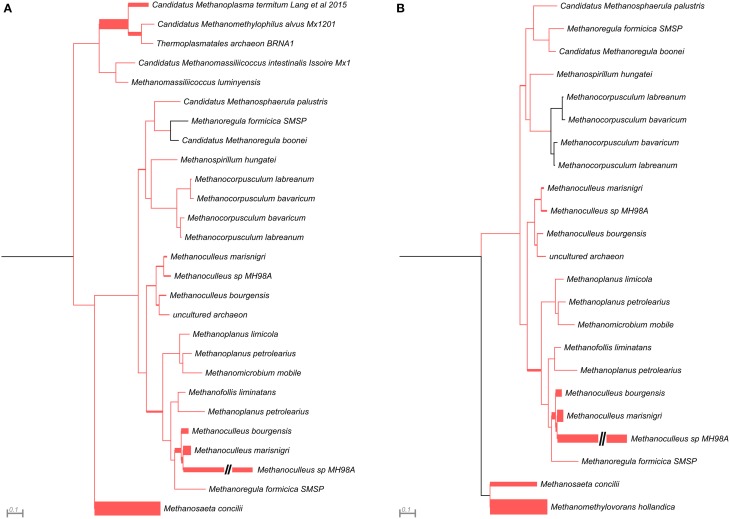
**Phylogenetic placement of *mcrB* amplicons from AD (A) and WD (B) samples**. The width of the red branches corresponds to the number of unique *mcrB* amplicon sequence reads in that particular branch (this can be either a leaf or node). The leaf for *Methanoculleus* sp. MH98A was shortened, as indicated by two slashes.

This analysis revealed that LMCRB/RMCRB primers, designed to the *mcrB* gene, amplified DNA fragments comprising sequences representing four methanogenic orders: *Methanobacteriales, Methanomassiliicoccales, Methanomicrobiales*, and *Methanosarcinales* (Figure 4). The dominant genus in both digesters was *Methanoculleus* spp. (48% for AD and 67% for WD), with *M. marisnigri* as the most abundant species (37 and 53%, respectively). This finding remains in good agreement with previous observations showing that the predominant order in biogas-producing microbial communities in anaerobic digesters is usually *Methanomicrobiales*, and the most abundant species is hydrogenotrophic *M. marisnigri* (Wirth et al., 2012). Moreover, in AD, 27% of sequences were classified as uncultured *Methanomassiliicoccales* (with 4% described as *Candidatus* Methanoplasma termitum) and 17% as *Methanosaeta concilli*. The second and third most abundant methanogens in WD were *Methanomethylovorans hollandica* (19%) and *Methanosaeta concilli* (6%), respectively (Figure 4).

**Figure 5:**
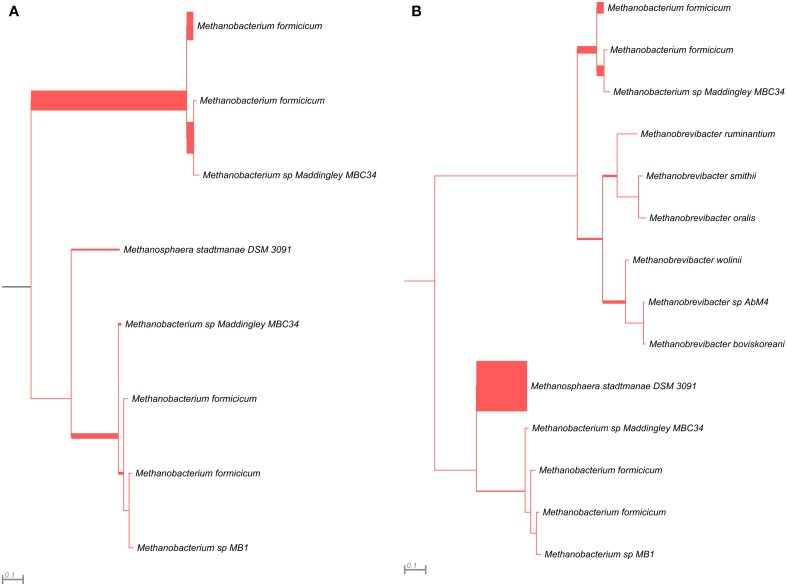
**Phylogenetic placement of *mcrG* amplicons from AD (A) and WD (B) samples**. The width of the red branches corresponds to the number of unique *mcrG* amplicon sequence reads in that particular branch (this can be either a leaf or node).

The *mcrG* gene fragments (amplified with primers LMCRG1/RMCRG1) comprised sequences representing five methanogenic orders: *Methanobacteriales, Methanococcales, Methanomicrobiales, Methanomassiliicoccales*, and *Methanosarcinales*. However, representatives of hydrogenotrophic *Methanobacteriales* were absolutely dominant in both digesters (Figure 5). The most abundant OTU_*mcrG*_ in AD was assigned to *Methanobacterium* spp. (97%) (with 7% mapped to *M. formicicum)*, while WD was dominated by *Methanosphaera stadtmanae* (54%) and *Methanobacterium* spp. (39%) (with 28% mapped to *M. formicicum)* and *Methanobrevibacter* spp. (5%) (Figure 5).

**Figure 6:**
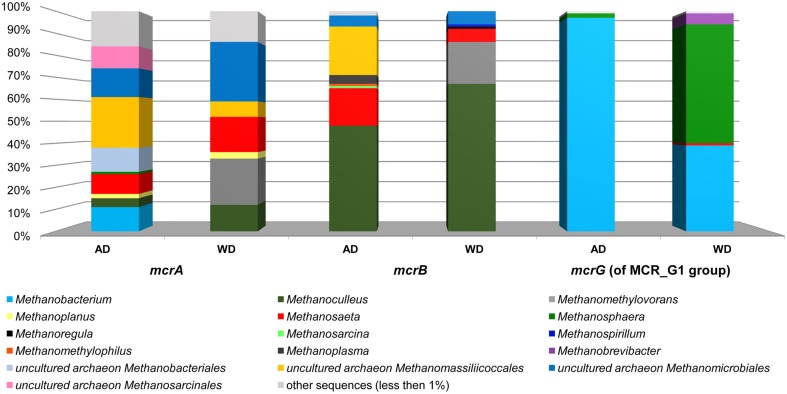
**Relative abundance of archaeal OTUs defined using the *mcrA* and *mcrB* and *mcrG* (of MCR_G1 group) gene fragments**. The bar chart shows the diversity of *Archaea* at the lowest reliable taxonomic level (mostly genus). AD, agricultural biogas plant anaerobic digester; WD, wastewater treatment plant anaerobic digester.

The above analysis revealed that primers LMCRB/RMCRB are highly specific for *mcrB* genes of methanogens. Therefore, similarly to the commonly employed *mcrA*-specific primers, they may be used for an overall characterization of the taxonomic structure of methanogenic communities. The application of both *mcrA* and *mcrB* molecular markers permits cross-checking and should give a deeper and more detailed insight into the taxonomic structure of various methanogenic communities. It is worth mentioning that the results obtained using the newly developed primers for *mcrB* were partially consistent with those obtained by *mcrA* analysis, and confirmed that the hydrogenotrophic pathway of methane synthesis is employed in the analyzed environments. Moreover, these results demonstrated the importance of the newly described seventh order of methanogenic *Methanomassiliicoccales* (Iino et al., 2013; Borrel et al., 2014) in the analyzed biogas digesters (Figure 6, Table S2).

The *mcrG* primers LMCRG1/RMCRG1 permitted the analysis of the minority of methanogenic *Archaea* that were not dominant in *mcrA*/*mcrB* analysis (except *Methanobacterium* for the *mcrA* marker). Therefore, the obtained results were not consistent with those obtained by *mcrA* and *mcrB* analyses. This is the consequence of the fact that the primers LMCRG1/RMCRG1 are specific only for the previously described MCR_G1 group of sequences (Figure S5) and their use could generate programmed bias (Figure 6, Table S2).

### Analysis of the selectivity of the *mtaB*- and *mtbA*-specific primers

In the course of this study, two other marker genes (*mtaB* and *mtbA*) specific for methylotrophic methanogens were selected and primer pairs developed. High-throughput sequencing of amplicons obtained using *mtaB* primers LMTAB/RMTAB detected sequences representing only two orders, *Methanosarcinales* and *Methanobacteriales*. In AD, 76% of sequences were assigned to *Methanosarcina* spp. [including *M. barkeri* (69%) and *M. mazei* (7%)] and 23% to *Methanosphaera stadtmanae*. Reactor WD was dominated by *M. hollandica* (94%), followed by *M. stadtmanae* (6%). In comparison, use of *mtbA*-specific primers LMTBA/RMTBA detected sequences mostly belonging to the *Methanosarcinales*, with two dominating species: *M. barkeri* (99%) in AD and *M. hollandica* (99%) in WD. Single sequences in WD and AD were assigned to *Halobacteriales* and *Methanomassiliicoccales*, respectively.

Sequencing of the *mtaB* and *mtbA* amplicons clearly indicated that in the analyzed digesters, *Methanosarcinales* are mainly responsible for the utilization of methylamines, while the conversion of methanol to methane is additionally performed by *M. stadtmanae* (of *Methanobacteriales*), which is consistent with previous studies (Fricke et al., 2006; Liu and Whitman, 2008).

## Conclusions

Four novel molecular markers were designed and tested for the detection and taxonomic analyses of methanogenic communities. Primers specific to the *mcrB* and *mcrG* genes (present in all methanogens), as well as the *mtaB* and *mtbA* genes, characteristic for methylotrophic methanogens, were developed. High-throughput sequencing of the amplicons obtained using these primers revealed their high specificity and indicated that these marker genes could be used for taxonomic profiling of methanogenic consortia.

The *mcrB* and *mcrG* molecular markers increased the resolution of high-throughput amplicon sequencing analyses of methanogenic communities that until now have only been investigated using the *mcrA* gene. The use of *mcrA, mcrB*, and *mcrG*, together with the 16S rRNA gene marker, should give a much broader overview of the taxonomic diversity of complex methanogenic communities. In addition, the analysis of two other marker genes (*mtaB* and *mtbA*) can provide an insight into the metabolic potential of the analyzed methanogens, since they permit the detection and analysis of an enigmatic group of methylotrophic methanogens, which are able to produce methane from methanol or methylamines.

### Conflict of interest statement

The authors declare that the research was conducted in the absence of any commercial or financial relationships that could be construed as a potential conflict of interest.

## Supplementary material

Figure S1 **Alignment of the conserved fragments of the *mcrB* genes of 36 methanogens used in the design of primers LMCRB and RMCRB**.

Figure S2 **Alignment of the conserved fragments of the *mcrG* genes (of MCR_G1 group) of 35 methanogens used in the design of primers LMCRG1 and RMCRG1**.

Figure S3 **Alignment of the conserved fragments of the *mtaB* genes of 26 methanogens used in the design of primers LMTAB and RMTAB**.

Figure S4 **Alignment of the conserved fragments of the *mtbA* genes of 13 methanogens used in the design of primers LMTBA and RMTBA**.

Figure S5 **Phylogenetic tree for *mcrG* nucleotide sequences (from NCBI database)**. The tree was constructed using the maximum-likelihood algorithm. Statistical support for the internal nodes was determined by 1000 bootstrap replicates and values of >50% are shown.
