# Supplementary figures and images for: Novel molecular markers for the detection of methanogens and phylogenetic analyses of methanogenic communities
Source: Front Microbiol. 2015 Jul 7;6:694. doi: 10.3389/fmicb.2015.00694 (PMC4493836; doi:10.3389/fmicb.2015.00694)

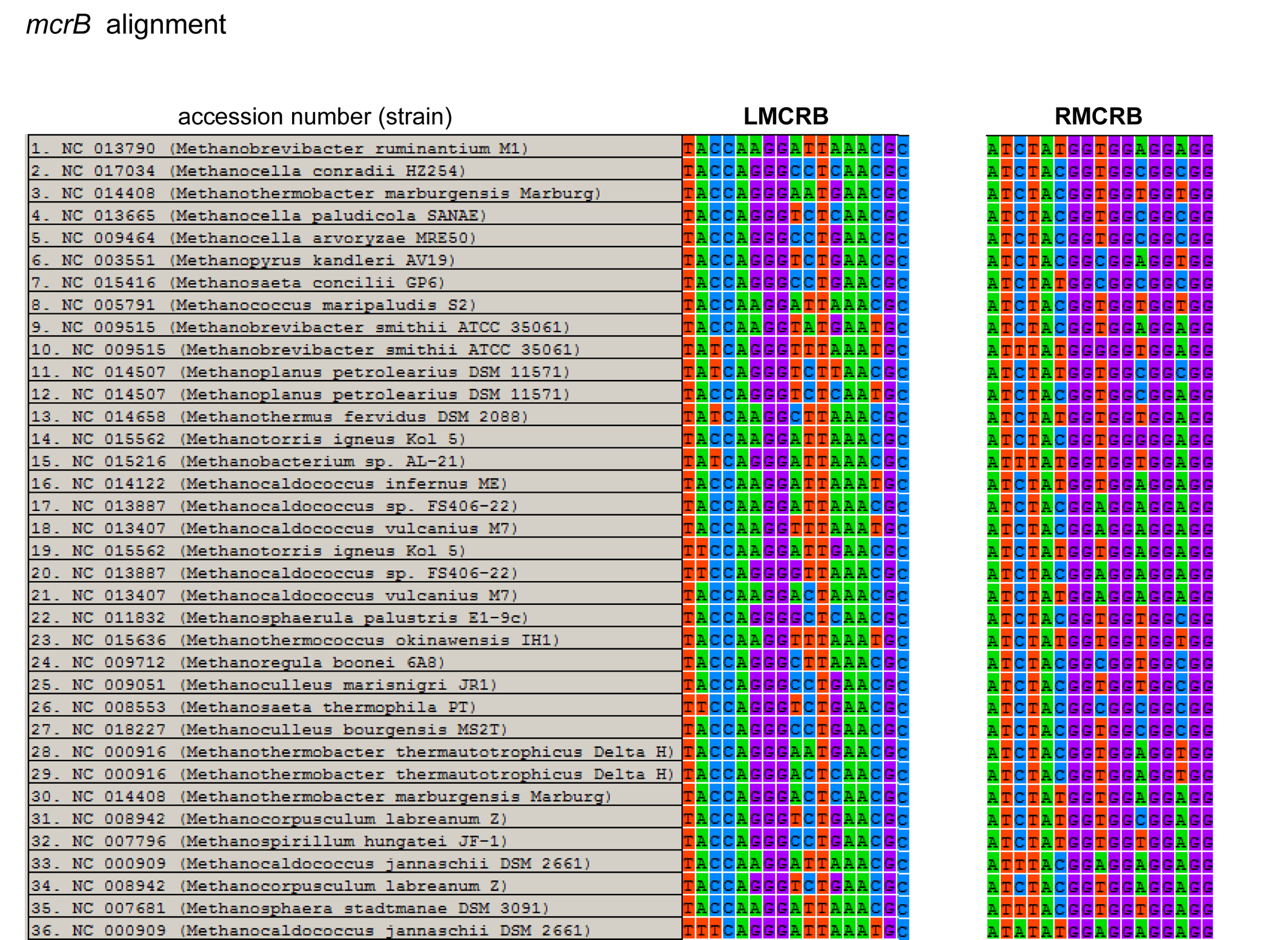

Supplement: Figure S1 — Alignment of the conserved fragments of the mcrB genes of 36 methanogens used in the design of primers LMCRB and RMCRB. [file Image1.TIF]

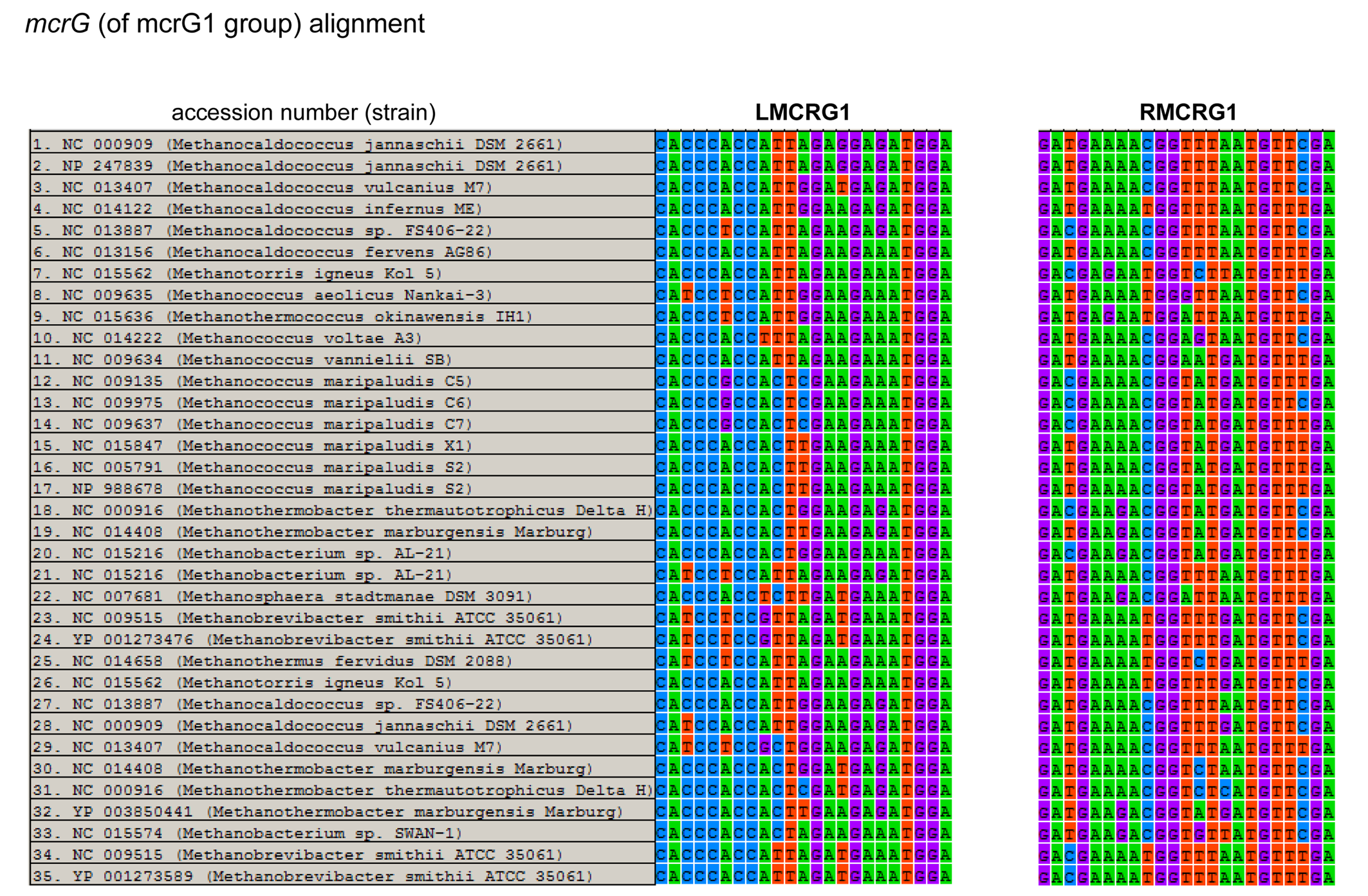

Supplement: Figure S2 — Alignment of the conserved fragments of the mcrG genes (of MCR_G1 group) of 35 methanogens used in the design of primers LMCRG1 and RMCRG1. [file Image2.TIF]

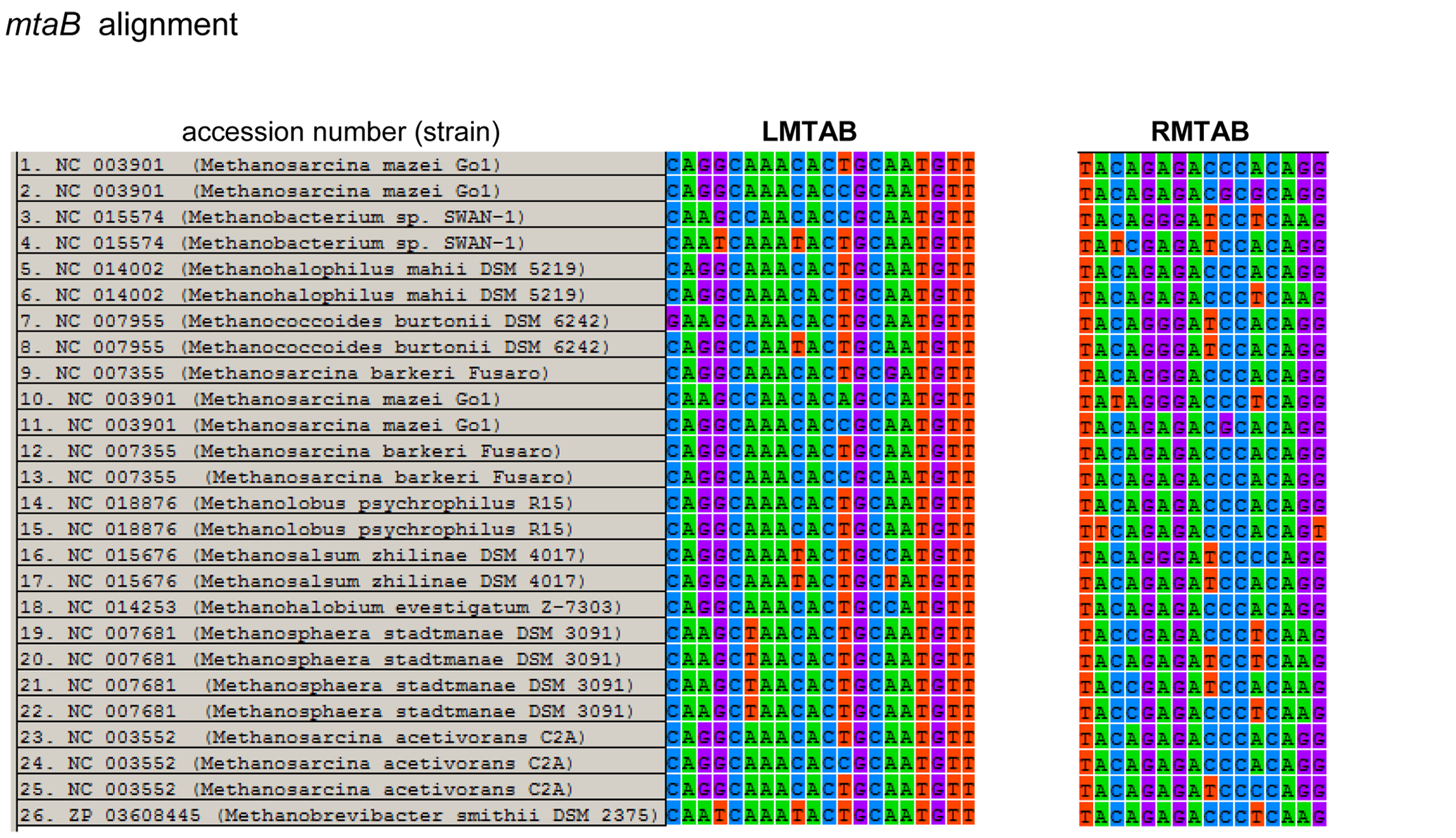

Supplement: Figure S3 — Alignment of the conserved fragments of the mtaB genes of 26 methanogens used in the design of primers LMTAB and RMTAB. [file Image3.TIF]

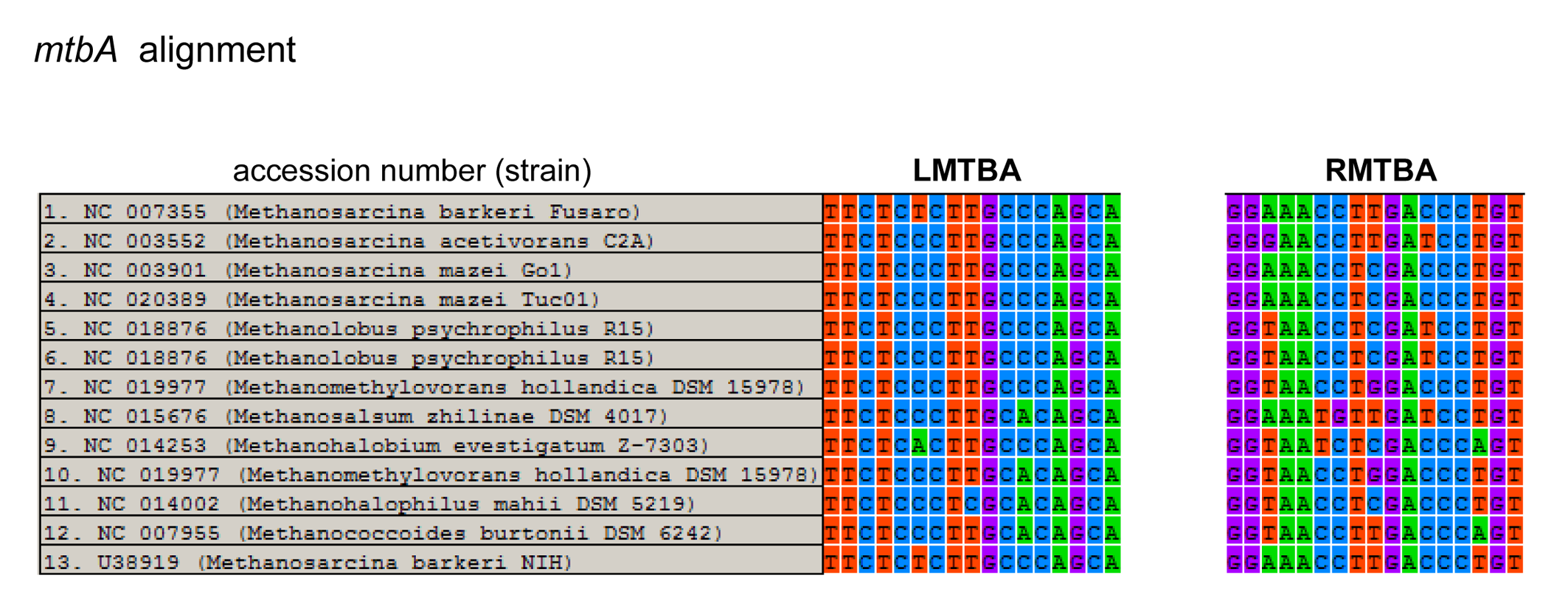

Supplement: Figure S4 — Alignment of the conserved fragments of the mtbA genes of 13 methanogens used in the design of primers LMTBA and RMTBA. [file Image4.TIF]

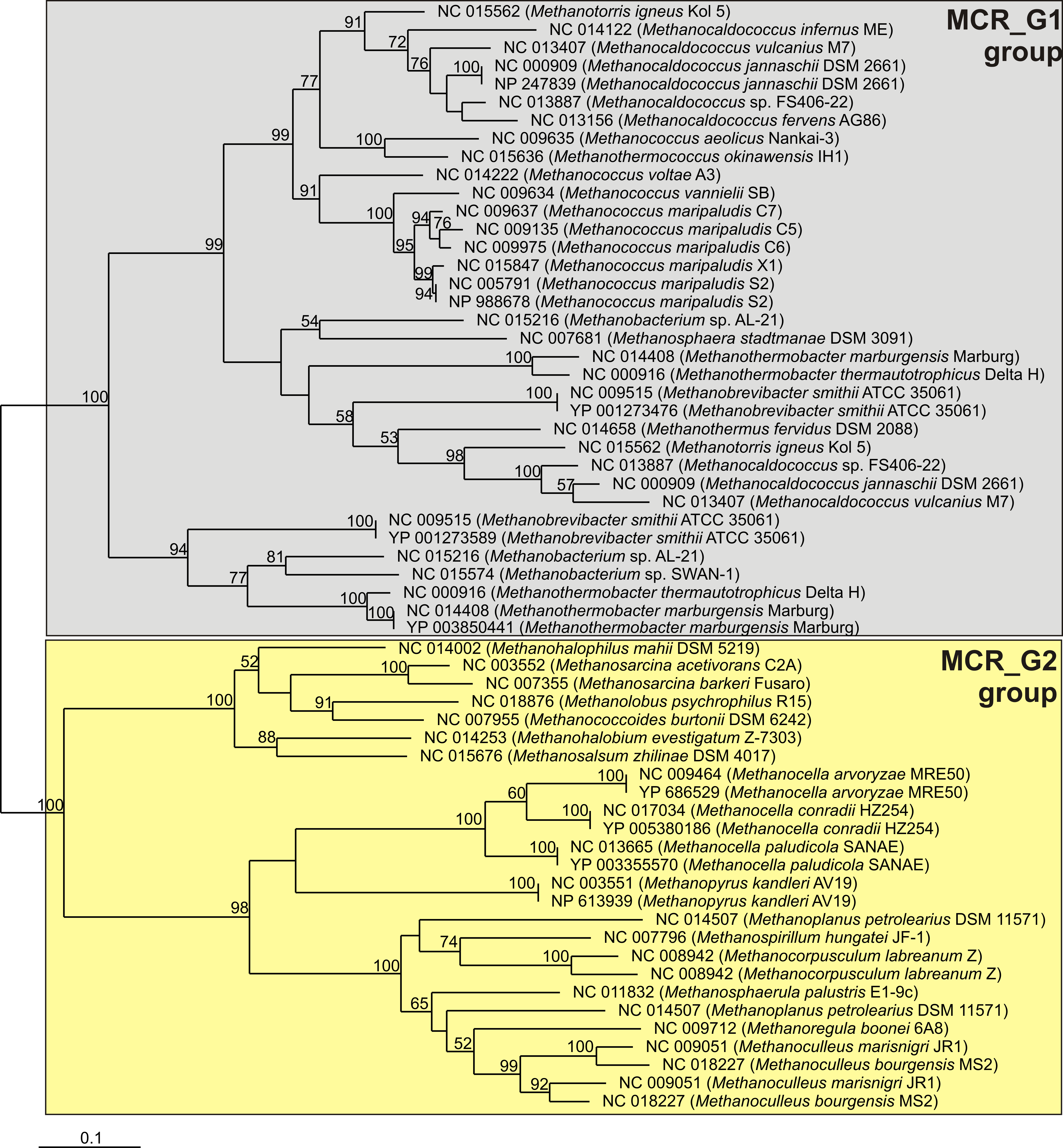

Supplement: Figure S5 — Phylogenetic tree for mcrG nucleotide sequences (from NCBI database). The tree was constructed using the maximum-likelihood algorithm. Statistical support for the internal nodes was determined by 1000 bootstrap replicates and values of >50% are shown. [file Image5.TIF]
