# Supplementary material for: Novel molecular markers for the detection of methanogens and phylogenetic analyses of methanogenic communities
Source: Front Microbiol. 2015 Jul 7;6:694. doi: 10.3389/fmicb.2015.00694 (PMC4493836; doi:10.3389/fmicb.2015.00694)
Supplement: Supplementary file 6 [file Table1.DOC]

| Primer pair | Product size range (bp) | Hits against 5274 complete microbial genomes (genus level) |
| --- | --- | --- |
| LMCRB/RMCRB | 386-389 | *Methanotorris*, *Methanothermus*, *Methanothermococcus*, *Methanothermobacter*, *Methanospirillum*, *Methanosphaerula*, *Methanosphaera*, *Methanosarcina*, *Methanosalsum*, *Methanosaeta*, *Methanoregula*, *Methanopyrus*, *Methanoplanus*, *Methanomethylovorans*, *Methanomassiliicoccus*, *Methanolobus*, *Methanohalobium*, *Methanoculleus*, *Methanocorpusculum*, *Methanococcus*, *Methanocella*, *Methanocaldococcus*, *Methanobrevibacter*, *Methanobacterium*, *Methanomethylophilus*  Non-methanogens: *Helicobacter*, *Thermoproteus* |
| LMCRG1/RMCRG1 | 353-356 | *Methanotorris*, *Methanothermus*, *Methanosphaera*, *Methanococcus*, *Methanocaldococcus*,  *Methanothermobacter,* Methanobrevibacter, *Methanobacterium*, |
| LMTBA/RMTBA | 413 | *Methanosarcina*, *Methanosalsum*, *Methanomethylovorans*, *Methanolobus*, *Methanohalophilus*, *Methanohalobium*, *Methanococcoides* |
| LMTAB/RMTAB | 436 | *Methanosphaera*, *Methanosarcina*, *Methanosalsum*, *Methanomethylovorans*, *Methanolobus*, *Methanohalophilus*, *Methanohalobium*, *Methanococcoides*, *Methanobrevibacter*, *Methanobacterium* |

**Table S1. Results of the *in silico* PCR analyses of developed primer pairs.**
