# Supplementary material for: Novel molecular markers for the detection of methanogens and phylogenetic analyses of methanogenic communities
Source: Front Microbiol. 2015 Jul 7;6:694. doi: 10.3389/fmicb.2015.00694 (PMC4493836; doi:10.3389/fmicb.2015.00694)
Supplement: Supplementary file 7 [file Table2.DOC]

|  | ***mcrA*** | | ***mcrB*** | | ***mcrG of MCR_G1 group*** | |
| --- | --- | --- | --- | --- | --- | --- |
|  | **AD** | **WD** | **AD** | **WD** | **AD** | **WD** |
| *Methanobacterium* | 11% | 0% | 0% | 0% | 97% | 39% |
| *Methanoculleus* | 4% | 12% | 48% | 67% | 0% | 0% |
| *Methanomethylovorans* | 0% | 21% | 0% | 19% | 0% | 0% |
| *Methanoplanus* | 2% | 3% | 0% | 0% | 0% | 0% |
| *Methanosaeta* | 9% | 16% | 17% | 6% | 0% | 1% |
| *Methanosphaera* | 1% | 0% | 0% | 0% | 2% | 54% |
| *Methanoregula* | 0% | 0% | 0% | 1% | 0% | 0% |
| *Methanosarcina* | 0% | 0% | 1% | 0% | 0% | 0% |
| *Methanospirillum* | 0% | 0% | 0% | 1% | 0% | 0% |
| *Methanomethylophilus* | 0% | 0% | 1% | 0% | 0% | 0% |
| *Methanoplasma* | 0% | 0% | 4% | 0% | 0% | 0% |
| *Methanobrevibacter* | 0% | 0% | 0% | 0% | 0% | 5% |
| uncultured archaeon *Methanobacteriales* | 11% | 0% | 0% | 0% | 0% | 0% |
| uncultured archaeon *Methanomassiliicoccales* | 23% | 7% | 22% | 0% | 0% | 0% |
| uncultured archaeon *Methanomicrobiales* | 13% | 27% | 5% | 6% | 0% | 0% |
| uncultured archaeon *Methanosarcinales* | 10% | 0% | 0% | 0% | 0% | 0% |
| other sequences (less then 1%) | 16% | 14% | 2% | 0% | 1% | 1% |

**Table S2. The summary of the abundance of the various methanogenic *Archaea* groups assessed by *mcrA*, *mcrB* and *mcrG* (of MCR_G1 cluster) genes analyses.**

* AD – agricultural biogas plant anaerobic digester; WD – wastewater treatment plant anaerobic digester.
